# Ethnic disparities in prevalence of chronic non-communicable diseases and its multimorbidity among older adults in rural southwest China

**DOI:** 10.1186/s12889-023-16161-1

**Published:** 2023-06-23

**Authors:** Ying-rong Du, Lan Liu, Yi Zhao, Jing-jing Huang, Allison Rabkin Golden, Le Cai

**Affiliations:** 1 The Third People’s Hospital of Kunming, Kunming, China; 2 School of Public Health, Kunming Medical University, Kunming, China; 3 The First Affiliated Hospital of Kunming Medical University, Kunming, China

**Keywords:** Chronic non-communicable diseases, Multimorbidity, Ethnicity, China

## Abstract

**Background:**

As the population ages, chronic non-communicable diseases (NCDs) multimorbidity has emerged as a major public health issue globally. This study examines ethnic disparities in prevalence of NCDs and its multimorbidity among rural southwest Chinese older adults.

**Methods:**

A cross-sectional survey was conducted in rural southwest population aged ≥ 60 years consisting of 5,642 consenting participants of Han and three ethnic minority groups (Dai, Ha Ni, and Bai). Information about participants’ demographic characteristics and lifestyle behaviors was obtained using a standard questionnaire. Anthropometric measurements including height, weight, and waist circumference, fasting blood sugar and blood pressure measurement, as well as post-bronchodilator spirometry test were recorded for each participant.

**Results:**

The age-standardized prevalence of five common chronic NCDs– hypertension, diabetes, coronary heart disease (CHD), stroke, chronic obstructive pulmonary disease (COPD) – and its multimorbidity was 72.8%, 15.9%, 4.0%, 10.0%, 9.8%, and 27.6%, respectively. Bai participants had both the highest overall and sex-specific prevalence rates of hypertension, diabetes, stroke, and COPD, whereas Han participants had the highest rates of CHD (*P* < 0.01). The results of multivariate logistic regression analysis indicated that female and older participants had a higher probability of chronic NCDs multimorbidity than their counterparts (*P* < 0.01). Bai ethnic minority participants were more likely to have NCDs multimorbidity while Ha Ni and Dai ethnic minority participants were less likely to have NCD multimorbidity relative to the Han participants (*P* < 0.05). Older adults with a higher level of education and family history of chronic NCDs, and who were also current smokers, current drinkers, obese, centrally obese, and physically inactive had a greater probability of developing chronic NCDs multimorbidity (*P* < 0.01).

**Conclusions:**

Ethnicity and individual demographic and lifestyle factors significantly impact prevalence of chronic NCDs multimorbidity. Future chronic NCDs prevention and control strategies must be tailored to address ethnicity, and culturally tailored lifestyle interventions may reduce the prevalence of chronic NCDs multimorbidity in rural southwest China.

## Introduction

Chronic non-communicable diseases (NCDs) remain the leading cause of morbidity and mortality worldwide, with 78% of NCDs-related mortality concentrated in low- and middle-income countries [1]. A key challenge in tackling NCDs is multimorbidity- or the co-existence of two or more chronic diseases in an individual [2]. As the population ages, multimorbidity has emerged as a major public health issue globally. It is projected that the world’s population aged 60 years and over will increase from 12% in 2015 to 22% by 2050 [3]. Older adults are at greater risk of suffering from multiple chronic diseases [4], and multimorbidity in the elderly population is associated with adverse health outcomes, including increased functional limitations, premature death, reduced quality of life, higher use of healthcare services, and higher probability of catastrophic health expenditure [5–7].

Over the last three decades, China has experienced rapid economic growth, urbanization, an aging population, and significant lifestyle changes. In parallel, the prevalence of NCDs such as stroke, hypertension, diabetes, coronary heart disease (CHD), and chronic obstructive pulmonary disease (COPD) have been on the rise. NCDs are now a major cause of premature death in China, accounting for > 90% of all adult deaths [8, 9]. Moreover, the proportion of people aged 60 years and over in China is forecast to rise from 18.7% in 2020 to 34.6% by 2050 [10]. As in many other countries, those who live to older age are more likely to experience multimorbidity of NCDs in China. Multimorbidity profoundly affects older adults’ wellbeing and increases great demand for healthcare utilization and social support, placing a significant strain on the Chinese health system [11, 12].

Previous studies exploring racial and ethnic disparities in prevalence of chronic NCDs multimorbidity worldwide revealed that the prevalence of chronic NCDs multimorbidity is likely to be higher in ethnic minority populations as compared to the majority population [13–15]. However, studies examining ethnic differences in prevalence of chronic NCDs multimorbidity, especially for older adults, are scarce in China, a populous, multiethnic nation with 56 distinct ethnicities and a Han ethnic majority accounting for 92% of the population. A deeper understanding of variation in chronic NCDs multimorbidity among the elderly and across ethnicities is critical for China to cope with its aging population and implement effective and culturally tailored chronic NCDs intervention strategies.

Yunnan Province is located in southwest China and contains the highest number of ethnic minority groups in China, with 25 ethnic minority groups residing in the province, 15 of which are found only in Yunnan. Each ethnic minority group in China has unique customs, lifestyle habits, and genetic backgrounds. Most of the ethnic minority group populations in Yunnan live in remote or mountainous areas, and on average are socioeconomically disadvantaged relative to the Han population.

Yunnan's population exceeds 47 million people, and ethnic minorities and older adults aged ≥ 60 years account for 33.12% and 14.91% of the region’s total population, respectively. However, there remains a dearth of analysis on prevalence and multimorbidity of chronic NCDs in this population. Thus, this study aimed to examine ethnic disparities in prevalence of five common chronic NCDs– hypertension, diabetes, stroke, CHD, and COPD– and its multimorbidity among the Han, Dai, Ha Ni, and Bai ethnic populations aged ≥ 60 years in rural southwest China.

## Methods

### Study area and population

A community-based cross-sectional health interview and examination survey was conducted in one majority Han-populated county and three unique ethnic minority regions of Yunnan Province. Data were collected between July 2021 and April 2022. Yunnan's 129 counties were divided into three strata based on geographic characteristics: valley region, dam region, and semi-mountainous or alpine region. One Han majority county was randomly chosen from the valley region, and one ethnic minority autonomous county was randomly chosen from each stratum, for a total of four counties. To select study participants ≥ 60 years from the four selected rural counties, a consistent three-stage stratified random sampling selection process was then employed. The following inclusion criteria were used: participants aged ≥ 60 years without cognitive dysfunction or inability to communicate with the interviewers, and residing in the selected village ≥ 5 years and willing to participate in the study. Details on this sampling technique can be found in previous research [16].

### Data collection and measurement

All consenting participants were interviewed in person, face-to-face, by trained interviewers. Self-reported data on demographic characteristics (age, sex, ethnicity, annual household income, and level of education), family history of the five studied chronic NCDs, and health behaviors (smoking and drinking habits, and physical activity) were obtained via an interviewer-administered structured, pre-tested questionnaire.

Anthropometric measurements, including height, weight, and waist circumference, three consecutive measurements of systolic and diastolic blood pressure (BP), fasting blood glucose (FBG), and three measurements of post-bronchodilator pulmonary function tests including pre- and post-bronchodilator forced expiratory volume in one second (FEV1) and forced vital capacity (FVC) were taken for all participants. Detailed descriptions on the data collection procedures have been previously reported and were guided by standardized protocols [16, 17].

### Definitions

Hypertension was defined as a mean systolic BP ≥ 140 mmHg or diastolic BP ≥ 90 mmHg, and/or use of antihypertensive medications to treat hypertension [18]. Diabetes mellitus was defined as a FBG value of ≥ 7.0 mmol/l (126 mg/dl), reported use of antidiabetic medications during the previous two weeks, or reported previous diagnosis of diabetes by a health professional [19]. COPD was defined as post-bronchodilator FEV1/FVC < 70%, according to the Global Initiative for Chronic Obstructive Lung Disease (GOLD) criteria [20]. Stroke and CHD were defined as self-report of a previous diagnosis by a physician. Multimorbidity was defined as the co-existence of two or more chronic NCDs in an individual, among the five studied diseases under investigation in this study [2].

Body mass index (BMI) was calculated as weight (kg) divided by height squared (m^2^). Participants with a BMI ≥ 28 kg/m^2^ were defined as obese. Central obesity was defined as a waist circumference of ≥ 90 cm in men and ≥ 80 cm in women, which aligns with WHO definitions for Asian adults [21]. Participants who had smoked at least 100 cigarettes over the course of their lives and smoked any form of tobacco product on a daily basis during the survey period were defined as current smokers, while participants who drank alcohol regularly on 12 or more days in one year preceding the survey were defined as current drinkers. Physical inactivity was defined as having engaged in moderate intensity exercise (for example, walking, running, cycling, and jogging) < 5 days a week for ≤ 30 min per day in the week prior to the survey, in accordance with WHO recommendations for physical activity in adults [22].

Illiteracy was defined as the inability to either read with understanding or to write a simple sentence about daily life. Annual household income was divided into two groups: low or high, with the median income used as the cut-off point to categorize all participants.

### Statistical analysis

All data analyses were conducted using R4.3.0 software. Data were analyzed with descriptive analysis techniques, chi-squared test, one-way ANOVA, and multivariate logistic regression. Categorical variables were presented as counts and percentages, while continuous variables were presented as mean ± standard deviation (SD). A chi-squared test for independence was used to compare categorical variables, while one-way ANOVA were conducted to analyze continuous measures across the ethnic groups. Age-standardized prevalence rates of five chronic NCDs were calculated by directly standardizing to the overall sample, and were computed as a percentage with a 95% confidence interval (CI). Multivariate logistic regression was used to analyze the association between individual demographic and health behaviors’ variables and the prevalence of chronic NCDs multimorbidity. The factors associated with chronic NCDs multimorbidity were initially assessed using univariable logistic regression analysis. Variables with *p* values < 0.05 in the univariable analysis were considered a candidate for the multivariate logistic regression. We have checked the assumptions for the requirements of multivariate logistic regression. Accordingly, the independence of variables was checked using the multi-collinearity test, and all variables were found independent of each other. Multivariate logistic regression model was then built to determine the adjusted OR with 95% CI using stepwise (backward selection, selection criteria: *p *value < 0.05). All statistical significance decisions were based on two-tailed *P* values of < 0.05.

## Results

The Han majority and three ethnic minority groups– Dai, Ha Ni, and Bai– were selected to participate in this study. Overall, 5,800 individuals aged ≥ 60 years were invited. Of these, 5,642 consented to participate, corresponding to a response rate of 97.3%.

Table 1 shows the demographic characteristics and mean values of BP, FBG, spirometry, and anthropometric measurements of the study population. In total, 2,718 (48.2%) of participants were male and 2,924 (51.8%) were female. Of these, 25.0%, 25.0%, 24.8%, and 25.1% of the population were Han, Dai, Ha Ni, and Bai ethnicities, respectively. Ha Ni ethnic minority participants had the lowest level of education, annual household income, mean height, mean weight, BMI, waist circumference, FBG, and prevalence of family history of NCDs, while they had the highest prevalence of current drinking and highest mean systolic and diastolic BP relative to the other three studied ethnicities (*P* < 0.01). The highest level of education was recorded in Han participants, while the Bai ethnic minority participants had the highest mean annual household income, FBG, prevalence of current smoking, obesity, central obesity, and physical inactivity (*P* < 0.01).

**Table 1:** Demographic characteristics and mean value of BP, FBG, spirometry, and anthropometric measurements of the study population

Characteristics	Han ethnic majority (*n* = 1413)	Dai ethnic minority (*n* = 1409)	Ha Ni ethnic minority (*n* = 1402)	Bai ethnic minority (*n* = 1418)	All (*n* = 5642)
Sex (n, %)					
Male	686 (48.5)	690 (49.0)	673 (48.0)	669 (47.2)	2718 (48.2)
Female	727 (51.5)	719 (51.0)	729 (52.0)	749 (52.8)	2924 (51.8)
Age (years, mean ± SD)	70.5 ± 6.3	69.2 ± 6.4	69.1 ± 6.5	70.5 ± 6.8	69.8 ± 6.5
Level of education (n, %)					
Illiterate	558 (39.5)	729 (51.7)	864 (61.6)**	581 (41.0)	2732 (48.4)
Primary (grade1-6) or higher	855 (60.5)	680 (48.3)	538 (38.4)	837 (59.0)	2910 (51.6)
Approximate annual household income (n, %)					
Low (< $960 US)	742 (52.5)	646 (45.8)	826 (58.9)	607(42.8)	2821 (50.0)
High (≥ $960 US)	671 (47.5)	763 (54.2)	576 (41.1)	811 (57.2)**	2821(50.0)
Current smoker (%)	295 (20.9)	262 (18.6)	334 (23.8)	458 (32.3)**	1349 (23.9)
Current drinker (%)	204 (14.4)	302 (21.4)	438 (31.2)**	196 (13.8)	1140 (20.2)
Obesity (%)	130 (9.2)	95 (5.3)	48 (3.4)	140 (9.9)**	393 (7.0)
Central obesity (%)	682 (48.3)	387 (27.5)	268 (19.1)	949 (66.9)**	2286 (40.5)
Physical inactivity (%)	778 (55.1)	583 (41.1)	690 (49.2)	878 (61.9)**	2929 (51.9)
Family history of hypertension	251 (17.8)	224 (15.9)	98 (7.0)**	244 (17.2)	817 (14.5)
Family history of diabetes	89 (6.3)	81 (5.7)	15 (1.1)**	86 (6.1)	271 (4.8)
Family history of CHD	123 (8.7)	83 (5.9)	8 (0.6)**	12 (0.8)	226 (4.0)
Family history of stroke	23 (1.6)	12 (0.9)	6 (0.4)**	18 (1.3)	59 (1.0)
Family history of COPD	124 (8.8)	81 (5.7)	15 (1.1)**	131 (9.2)	351 (6.2)
Family history of NCDs (%)	312 (22.1)	251(17.8)	109 (7.8)**	300 (21.2)	972 (17.2)
Height (cm, mean ± SD)	157.2 ± 8.7	157.0 ± 7.9	153.0 ± 8.1	158.7** ± 8.3	156.5 ± 8.5
Weight (kg, mean ± SD)	56.6 ± 10.9	53.8 ± 10.4	50.5 ± 9.4	59.9** ± 10.1	55.2 ± 10.8
BMI (kg/m2, mean ± SD)	22.8 ± 3.7	21.8 ± 3.7	21.5 ± 3.2	23.7** ± 3.4	22.5 ± 3.6
Waist circumference (cm, mean ± SD)	81.1 ± 10.1	75.6 ± 9.7	73.8 ± 9.3	85.8** ± 9.2	79.1 ± 10.2
Systolic BP (mm Hg, mean ± SD)	139 ± 22	136 ± 19	144** ± 23	142 ± 21	140 ± 21
Diastolic BP (mm Hg, mean ± SD)	83 ± 12	81 ± 10	86** ± 12	84 ± 11	83 ± 12
FBG (mmol/l, mean ± SD)	5.7 ± 1.4	5.7 ± 1.1	5.3 ± 1.0	6.0** ± 1.8	5.7 ± 1.4
Spirometry † ( mean ± SD)					
FEV1, L	1.7 ± 0.5	1.7 ± 0.5	1.7 ± 0.5	1.5 ± 0.5	1.7 ± 0.5
FVC, L	2.1 ± 0.6	2.1 ± 0.6	2.2 ± 0.6	1.9 ± 0.6	2.1 ± 0.6
FEV1/FVC, %	80.1 ± 8.5	81.5 ± 9.6	78.6 ± 8.3	77.3* ± 8.6	79.4 ± 9.1

*BMI* body mass index, *BP* blood pressure, *FBG* fasting blood glucose, *SD* standard deviation

^*^ *P* < 0.05, ** *P* < 0.01, †pre-bronchodilator

Table 2 presents age-standardized prevalence and multimorbidity of five chronic NCDs by ethnicity among the study population. 79.8% of the older adults suffered from at least one of the five studied non-communicable chronic conditions. The overall prevalence of hypertension, diabetes, CHD, stroke, COPD, and multimorbidity of the five studied NCDs among those surveyed were 72.8%, 15.9%, 4.0%, 10.0%, 9.8%, and 27.6%, respectively. Bai participants had both the highest overall and sex-specific prevalence rates of hypertension, diabetes, stroke, COPD, and chronic NCDs multimorbidity (*P* < 0.01), while Han participants had both highest overall and sex-specific prevalence rate of CHD (*P* < 0.01). In all four studied ethnicities, males had a higher prevalence of stroke and markedly higher prevalence of COPD than females (*P* < 0.01), while females had higher prevalence of multimorbidity of chronic NCDs than males (*P* < 0.01). Prevalence of hypertension and CHD did not differ by sex (*P *> 0.05). Ha Ni female participants had higher prevalence of diabetes than males (*P* < 0.01).

**Table 2:** Distribution of age-adjusted prevalence and multimorbidity of five chronic NCDs among rural adults aged ≥ 60 years by ethnicity and sex in rural southwest China

Ethnicity	Hypertension % (95% CI)	Diabetes% (95% CI)	CHD % (95% CI)	Stroke% (95% CI)	COPD% (95% CI)	Number of NCDs % (95% CI)		
Han ethnic majority								
Male	61.4 (57.7, 64.9)	11.7 (9.5, 14.3)	6.1^b^ (4.6, 8.2)	9.0^c^ (7.1, 11.4)	10.8^d^ (8.7, 13.3)	29.0 (25.7, 32.5)	71.0 (67.5, 74.3)	20.2 (17.6, 24.6)
Female	67.4 (63.9, 70.7)	12.2 (10.1, 14.8)	6.6^b^ (5.0, 8.6)	7.2 (5.5, 9.3)	4.0 (2.8, 5.7)	27.6 (23.5, 29.9)	72.4(70.1, 76.5)	24.3^c^ (20.5, 27.8)
All	64.5 (61.9, 66.9)	12.0 (10.4, 13.8)	6.4^b^ (5.2, 7.8)	8.1 (6.8, 9.6)	7.3 (6.1, 8.8)	27.7 (25.5, 30.1)	72.3 (69.9, 74.5)	22.2 (20.3, 24.7)
Dai ethnic minority								
Male	63.9 (60.3, 67.4)	7.1 (5.4, 9.3)	3.8 (2.6, 5.5)	6.1^d^ (4.5, 8.1)	11.0^d^ (8.9, 13.6)	27.5 (24.3, 31.0)	72.5 (69.0,75.7)	12.0 (9.9, 14.7)
Female	62.6 (59.0, 66.1)	8.1 (6.3, 10.3)	2.8(1.8, 4.3)	2.8 (1.8, 4.3)	4.9 (3.5, 6.7)	31.9^c^ (28.6, 35.3)	68.2 (64.7, 71.5)	16.2^d^ (13.9, 19.5)
All	63.2 (60.7, 65.7)	7.6 (6.3, 9.1)	3.3 (2.5, 4.3)	4.4 (3.5, 5.6)	7.9 (6.6, 9.4)	29.7 (27.4, 32.2)	70.3 (67.8, 72.6)	14.1 (12.5, 16.2)
Ha Ni ethnic minority								
Male	66.0 (62.3, 69.5)	6.4^c^ (4.8, 8.5)	3.3 (2.2, 4.9)	3.9^c^ (2.7, 5.6)	12.0^d^ (9.8, 14.7)	27.6(24.2, 31.1)	72.4 (68.9, 75.6)	9.7 (8.2, 12.6)
Female	61.0 (57.5, 64.5)	3.8 (2.7, 5.5)	4.0 (2.8, 5.7)	2.5 (1.6, 3.9)	5.9 (4.4, 7.9)	33.2^a^ (29.9, 36.7)	66.8 (63.3, 70.1)	16.1^d^ (13.9, 19.5)
All	63.4 (60.9, 65.9)	5.1 (4.0, 6.3)	3.6 (2.8, 4.8)	3.1 (2.4, 4.)	8.8 (7.5, 10.4)	30.5 (28.2, 33.0)	69.5 (67.0, 71.8)	12.8 (11.5, 15.1)
Bai ethnic minority								
Male	68.3^b^(64.7, 71.7)	15.3^b^ (12.7, 18.2)	4.4 (3.3, 7.4)	10.5^bc^(8.4, 13.0)	17.0^d^ (14.4, 20.1)	21.7 (18.7, 25.0)	78.3 (75.1, 85.3)	24.7^bd^ (22.0, 28.2)
Female	76.8^b^ (73.6, 79.7)	16.6^b^(14.1, 19.4)	3.7(1.7, 4.8)	9.6^bc^ (7.7, 11.9)	3.7 (2.3, 4.9)	18.8 (16.2, 21.8)	81.2 (78.2, 83.8)	30.8^bd^ (28.0, 35.0)
All	72.8^b^ (70.4, 75.0)	15.9^b^ (14.1, 17.9)	4.0 (3.1, 5.1)	10.0^bc^ (8.6, 11.7)	9.8^b^ (8.4, 11.5)	20.2 (18.2, 22.3)	79.8 (77.7, 81.8)	27.6^bd^(25.7, 30.4)

^a^*p* < 0.05, ^b^*p* < 0.01, comparisons among different ethnic populations; ^c^*p* < 0.05, ^d^*p* < 0.01, comparisons between different sexes

**Fig. 1:**
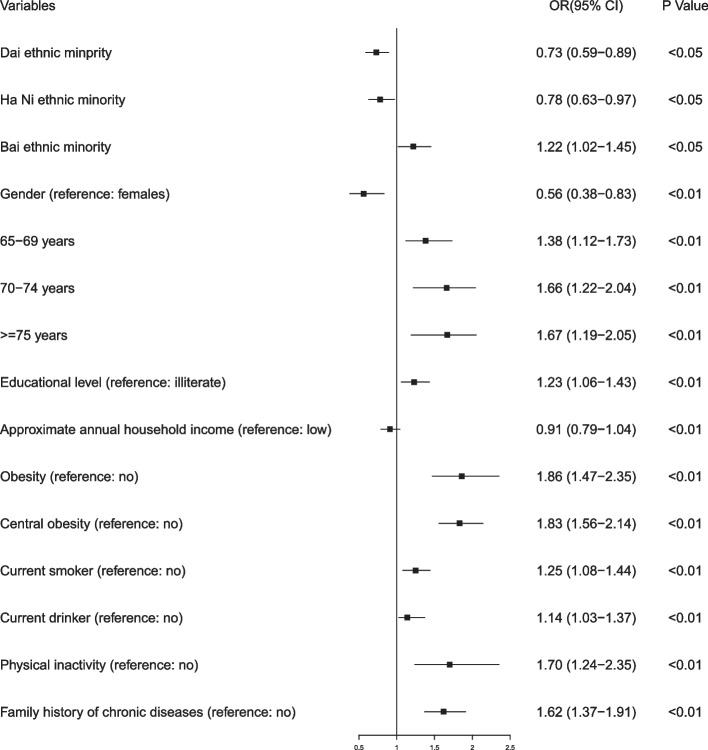
Forest plot for multi-variable logistic regression for multimorbidity of five chronic NCDs

Table 3 and Fig. 1 display results of multivariate logistic regression analysis. Females and older participants had a higher probability of multimorbidity of chronic NCDs than their counterparts (*P* < 0.01). Bai ethnic minority older adults were more likely to have NCDs multimorbidity while Ha Ni and Dai ethnic minority older adults were less likely to have NCDs multimorbidity relative to Han majority counterparts (*P* < 0.05). Older adults with a higher level of education and family history of chronic NCDs, older adults who were current smokers and current drinkers, obese, centrally obese, and physically inactive older adults had a greater probability of multimorbidity of chronic NCDs (*P* < 0.01).

**Table 3:** Odds ratios (OR) and 95% confidence intervals (CI) for multi-variable logistic regression for multimorbidity of five chronic NCDs

Characteristic	Multimorbidity (reference: no)	
	Odds ratio (OR)	95% CI
Ethnicity (reference: Han ethnic majority)		
Dai ethnic minority	0.73*	[0.59–0.89]
Ha Ni ethnic minority	0.78*	[0.63–0.97]
Bai ethnic minority	1.22*	[1.02–1.45]
Gender (reference: females)	0.56**	[0.38–0.83]
Age(reference:60-64 years)		
65–69 years	1.38**	[1.12–1.73]
70–74 years	1.66**	[1.22–2.04]
≥ 75 years	1.67**	[1.19–2.05]
Educational level (reference: illiterate)	1.23**	[1.06–1.43]
Approximate annual household income (reference: low)	0.91	[0.79–1.04]
Obesity (reference: no)	1.86**	[1.47–2.35]
Central obesity (reference: no)	1.83**	[1.56–2.14]
Current smoker (reference: no)	1.25**	[1.08–1.44]
Current drinker (reference: no)	1.14**	[1.03–1.37]
Physical inactivity (reference: no)	1.70**	[1.24–2.35]
Family history of chronic diseases (reference: no)	1.62**	[1.37–1.91]

^*^P < 0.05, ** P < 0.01

## Discussion

The findings indicate the prevalence of five studied chronic NCDs and its multimorbidity varied by ethnicity in rural southwest China, and chronic NCDs multimorbidity was independently associated with both individual demographic and lifestyle factors.

The present study also revealed significant ethnic differences in lifestyle behaviors across the four studied ethnicities. Bai ethnic minority older adults had the highest prevalence rates of current smoking, obesity, central obesity and physical inactivity, while Ha Ni participants had the highest rate of current drinking. Correspondingly, Bai ethnic minority older adults also had the highest prevalence rates of hypertension, diabetes, stroke, COPD, and NCDs multimorbidity compared to the other three minority groups. The findings in this way highlight a pressing need for lifestyle interventions to head off an emerging epidemic of chronic NCDs in ethnic minority populations.

Our study indicated that CHD is more common in Han majority older adults than the other three ethnic minority groups. Such ethnic variations in prevalence of CHD were also observed in studies conducted in western countries [23, 24]. This possibly results from the fact that Han participants had the highest prevalence of family history of CHD (8.7%) among the four studied ethnic groups. Moreover, the development of CHD is known to be dependent upon a complex interplay of both genetic and environmental factors [25]. Further investigation is needed to examine the exact nature of the association between ethnicity and CHD.

Males had a markedly higher prevalence rate of spirometry-defined COPD than females across all four ethnicities studied. This finding accords with many previous studies conducted in both developed and developing countries [26, 27], and may result from the fact that males smoked significantly more than females in the participant population (66.9% vs. 1.0%) as previous studies have demonstrated that tobacco smoking is one of the major contributing causes of COPD in low and middle-income countries [28, 29]. Our findings present a challenge for local governments to take community-based COPD prevention strategies to further control COPD focused particularly on males.

Overall, 27.6% of older adult participants in the present study suffered from NCDs multimorbidity, a rate lower than that found in a previous Chinese study conducted in Shangdong province (34.71%) [30] and Shanxi province (30.3%) [31], but higher than in India (24.0%) [32]. However, comparison of NCDs multimorbidity studies is difficult as findings may be heavily influenced by the number and types of chronic conditions included and analyzed.

In this study, Bai ethnic minority older adults were more likely to have NCDs multimorbidity while Ha Ni and Dai ethnic minority older adults were less likely to have NCDs multimorbidity relative to Han majority counterparts. The considerable ethnic differences in NCDs multimorbidity prevalence possibly arise from ethnic minority groups’ differing cultural practices and customs and lifestyle habits, differing genetic structure shaped by their marriage patterns, and many ethnic minority groups in China are socioeconomically disadvantaged with less access to healthcare relative to the Han population [16, 33]. The ethnic differences in NCDs multimorbidity indicate ethnicity is a key determinant for NCDs multimorbidity, and ethnicity should be a consideration when developing NCDs multimorbidity prevention and intervention strategies in rural southwest China.

Female older adults were more likely to have NCDs multimorbidity and prevalence of multimorbidity increase with age. This result is in line with many previous studies both in and outside China [4, 11, 30, 31]. Furthermore, obese or centrally obese older adults, current smokers, current drinkers, physically inactive older adults, and older adults with family history of chronic NCDs had greater odds of NCDs multimorbidity. Being obese or centrally obese, smoking, drinking alcohol, physical inactivity, and having a family history of chronic NCDs are all well-established major risk factors for NCDs multimorbidity [13, 14, 30–32]. These results thereby underscore comprehensive lifestyle interventions are essential for prevention and management of NCDs multimorbidity.

There is growing evidence that people with a lower level of education and income are more likely to have NCDs multimorbidity across the globe [11, 34, 35]. However, our study did not find this to be true in the communities we surveyed. Instead, in our study, individual household income had no association with NCDs multimorbidity, while older adults with a higher level of education had a greater risk for developing NCDs multimorbidity. The causes behind this differing effect of income on presence of NCDs multimorbidity require further research. The positive relationship between individual education level and NCDs multimorbidity in this study possibly results from the fact that more educated individuals are likely to have better health literacy and a higher use of healthcare services and thus, are more likely to be diagnosed with NCDs than their lower education counterparts.

There are several limitations to our study. First, the study was cross-sectional in design, so causal relationships cannot be determined. Second, haemoglobin A1C and oral glucose tolerance were not measured, and diagnose of diabetes was solely based on FBG test, which could have caused an underestimation of the prevalence of diabetes. Third, stroke and CHD were defined based on self-reporting of the physician’s diagnosis, potential misclassification is possible. Moreover, due to the lack of medical knowledge among Chinese rural residents, some patients without access to quality healthcare may not be aware of their health status, the prevalence of target diseases might be underestimated. Fourth, multimorbidity in our study was based on only five chronic NCDs; the prevalence of multimorbidity in our study may be underestimated. Finally, the present findings were based on a study of random sampling of four ethnic groups, limiting the ability to generalize the results to other ethnic groups in Yunnan.

## Conclusion

The present study indicates chronic NCDs and its multimorbidity is a serious public health challenge in rural southwest China, and there were significant associations between ethnicity, individual demographic, and lifestyle factors and the prevalence of chronic NCDs multimorbidity. The results of our study underscore the necessity of considering ethnicity as a factor in future chronic NCDs prevention and management strategies. Although it is possible that providing culturally tailored lifestyle interventions might help to reduce the prevalence of chronic NCDs multimorbidity in rural southwest China, more investigations are needed.

## Data Availability

The datasets used and/or analyzed during the current study is available from the corresponding author on reasonable request.

## Abbreviations

BMI: Body mass index
BP: Blood pressure
CHD: Coronary heart disease
CI: Confidence interval
COPD: Chronic obstructive pulmonary disease
FBG: Fasting blood glucose
FEV1: Pre- and post-bronchodilator forced expiratory volume in one second
FVC: Forced vital capacity
GOLD: Chronic obstructive lung disease
NCDs: Chronic non-communicable diseases
OR: Odds ratios
SD: Standard deviation
WHO: World health organization
